# Optimal Antithrombotic Strategies in Cardiogenic Shock

**DOI:** 10.3390/jcm13010277

**Published:** 2024-01-03

**Authors:** Michal Droppa, Tobias Geisler

**Affiliations:** Department of Cardiology and Angiology, University Hospital of Tuebingen, 72076 Tuebingen, Germany

**Keywords:** cardiogenic shock, antithrombotic treatment, mechanical circulatory support, thrombosis, bleeding

## Abstract

Cardiogenic shock (CS) represents a critical condition with a high mortality rate. The most common cause of CS is coronary artery disease, and patients typically present with myocardial infarction, necessitating immediate treatment through percutaneous coronary intervention (PCI) and often requiring mechanical circulatory support. CS is associated with a prothrombotic situation, while on the other hand, there is often a significant risk of bleeding. This dual challenge complicates the selection of an optimal antithrombotic strategy. The choice of antithrombotic agents must be personalized, taking into consideration all relevant conditions. Repeated risk assessment, therapeutic monitoring, and adjusting antithrombotic therapy are mandatory in these patients. This review article aims to provide an overview of the current evidence and practical guidance on antithrombotic strategies in the context of CS.

## 1. Introduction

Cardiogenic shock (CS) is a critical condition with high mortality. It most commonly occurs in the setting of acute coronary syndrome (ACS) and requires immediate treatment with percutaneous coronary intervention (PCI). Despite optimal interventional therapy, CS is associated with high morbidity and mortality. It is often accompanied by a prothrombotic situation, making effective adjunctive antithrombotic therapy a cornerstone of therapy in this high-risk condition. However, challenges exist with regard to administration, pharmacodynamics, and the combination of anticoagulation and antiplatelet therapy. During CS, there is an imbalance between required and available organ perfusion. As a result, drug absorption and metabolism may be altered, leading to reduced efficacy and the risk of thrombotic complications. In addition, CS patients are threatened by major bleeding events, in particular those undergoing mechanical support, making a dynamic, repeated risk assessment and adjustment of antithrombotic therapy mandatory. Therefore, the choice of an appropriate antithrombotic strategy must be individualized, taking all conditions into account. This review article will give an overview of current evidence and practical guidance on antithrombotic strategies in CS.

## 2. Definition and Pathophysiology

**Figure 1:**
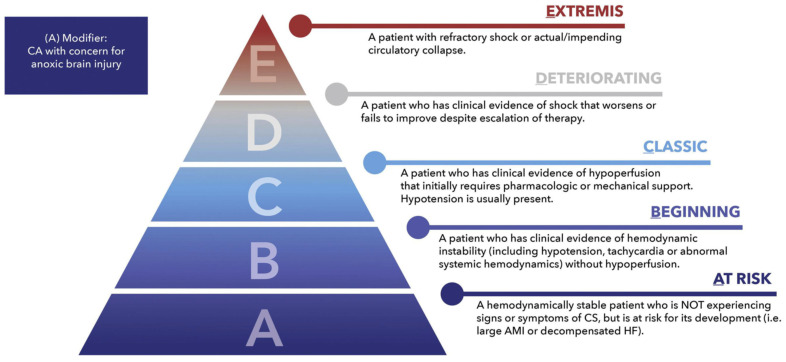
SCAI SHOCK Stage Classification according to [1]. AMI—acute myocardial infarction; CA—cardiac arrest; CS—cardiogenic shock; HF—heart failure; SCAI—Society for Cardiovascular Angiography and Interventions.

The current definition and classification of the CS are based on the SCAI Shock Expert Consensus [1]. It emphasizes the dynamic process of the CS and includes five stages (Figure 1).

Stages A and B are patients “at risk” and patients with beginning hemodynamic instability without signs of systemic hypoperfusion. The “classic” cardiogenic shock includes patients from stage C; the patients have evidence of systemic and organ hypoperfusion. The hemodynamics of patients with CS are characterized by hypotension (systolic blood pressure < 90 mmHg, or a mean arterial pressure < 60 mmHg, or a drop from baseline of >30 mmHg). Manifest CS requires pharmacological or mechanical intervention beyond volume resuscitation. Other hemodynamic features include a low cardiac index (<2.2 L/min/m^2^) and high pulmonary capillary wedge pressure (>15 mmHg). Verification of organ hypoperfusion relies on clinical examination and laboratory findings (elevated serum lactate, BNP, renal, and hepatic markers). Various conditions, such as myocarditis, cardiomyopathies, valvular disease, pericardial tamponade, pulmonary embolism, or arrhythmias, can lead to cardiogenic shock. However, the most common cause of CS remains coronary artery disease, which usually manifests as ACS. Immediate revascularization is the standard therapy for CS in patients with STEMI and NSTEMI and can significantly improve the prognosis. PCI with coronary stent implantation is usually performed to improve the critical coronary perfusion, so the antithrombic strategy is of utmost importance and will be discussed in the following review.

There are several important pathophysiologic changes during CS that may influence the pharmacokinetics of the antithrombotic agents used. Organ hypoperfusion may delay drug absorption from the gastrointestinal tract. Underlying pathophysiologic mechanisms include decreased gastrointestinal motility, delayed gastric emptying, and diminished absorption. Furthermore, since CS patients are often under mechanical ventilation, oral medications must be administered via a nasogastric tube, further prolonging drug administration. There are also pharmacokinetic changes in drug metabolism. Hepatic dysfunction in CS may reduce the activation of prodrugs to active metabolites, diminishing drug efficacy. This becomes particularly relevant for oral P2Y_12_ receptor inhibitors that are activated by liver enzymes. On the other hand, hepatic elimination of drugs may be reduced, which may lead to the accumulation of substances such as oral anticoagulants (OAC), thus increasing the risk of bleeding. Similar to liver dysfunction, acute kidney injury often accompanies CS. This can lead to the accumulation of renally excreted drugs. Renal replacement therapy may also affect drug elimination, depending on the method used. Therefore, more frequent drug monitoring may be required in CS. Mild therapeutic hypothermia may also affect the pharmacokinetics of antithrombotic drugs, most likely due to impaired prodrug activation. Mechanical circulatory support with extracorporeal membrane oxygenation (ECMO) may induce sequestration of some drugs and decrease drug clearance. Drug–drug interactions may also be relevant for patients with CS. Mechanically ventilated patients require analgesia/sedation with morphine derivatives, which reduce the effect of oral antiplatelet drugs mainly due to prolonged absorption.

Similar to other disease conditions, there is a relevant overlap of bleeding and thrombotic risk factors. Risk factors can be generally divided into patient-dependent and treatment-dependent risk factors (graphical abstract). Rates for major and fatal bleeding in CS have been reported at rates between 10–20% and 3–5%, respectively, depending on bleeding classification and location (i.e., access versus non-access site bleedings) [2,3]. In the CULPPRIT-SHOCK trial, bleeding events complicated the course of CS and were associated with increased mortality. Sepsis, peripheral ischemic complications, new atrial fibrillation, and treatment with active mechanical support by ECMO or Impella were risk factors for bleeding complications [4].

## 3. Characteristics of Antithrombotic Substances

The pharmacological properties of different antithrombotic substances and special aspects of CS are summarized in Table 1.

### 3.1. Aspirin

Aspirin (acetylsalicylic acid) is an irreversible inhibitor of cyclooxygenase-1. Through irreversible acetylation of this enzyme, the formation of thromboxane-A2 in platelets is blocked. The effect persists for the life-time of platelets in the circulation (7–10 days). Aspirin can be administered per os or intravenously. The usual loading dose in aspirin-naïve patients is 150–300 mg. Aspirin is given in doses of 75–100 mg/day.

Aspirin is the standard first-line therapy for patients with ACS. According to ESC guidelines, a loading dose of 150–300 mg orally or 75–250 mg intravenously should be given as soon as possible [5]. There are no large randomized controlled trials comparing intravenous and oral aspirin. However, pharmacological data show worse outcomes with aspirin undertreatment [6] and better platelet inhibition with intravenous administration [7]. Given the unfavorable prognosis of CS and the importance of a rapid antithrombotic effect, it seems reasonable to administer aspirin intravenously.

### 3.2. P2Y_12_ Receptor Inhibitors

#### 3.2.1. Clopidogrel

Clopidogrel is a thienopyridine prodrug that irreversibly inhibits the platelet P2Y_12_-receptor after biotransformation in the liver. It prevents ADP-mediated platelet activation. Hepatic activation of clopidogrel requires two steps of activation by cytochrome P450. Therefore, the therapeutic effect of clopidogrel occurs with a delay of 30–60 min after oral administration of the loading dose (usually 600 mg). A significant limitation of clopidogrel is also the inter-individual variability of its metabolism, resulting in a low clopidogrel response to therapy in a relevant number of patients [26].

#### 3.2.2. Prasugrel

Prasugrel is an antiplatelet agent that acts similarly to clopidogrel by irreversibly inhibiting the P2Y_12_ receptor and thus the ADP-dependent pathway of platelet aggregation. This thienopyridine derivative is also a prodrug, but has a shorter one-step biotransformation and therefore a faster onset of antiplatelet activity than clopidogrel [27].

#### 3.2.3. Ticagrelor

Ticagrelor is a nucleoside analogue that reversibly blocks the P2Y_12_ receptor at a different binding site than clopidogrel and prasugrel. Unlike thienopyridines, it does not require bioactivation. It has a rapid onset of action of about 30 min.

There are no large randomized trials comparing the use of different P2Y_12_ receptor inhibitors in the population of patients with CS. A retrospective analysis of the IABP-Shock II trial showed no difference in mortality between clopidogrel, prasugrel, and ticagrelor, with ticagrelor being associated with the lowest bleeding risk [8]. Two small retrospective studies with clinical endpoints (stent thrombosis, mortality) comparing clopidogrel and prasugrel/ticagrelor in the setting of cardiac arrest and mild therapeutic hypothermia have been published with conflicting results [9,10]. In three pharmacological studies, prasugrel and ticagrelor showed significantly better platelet inhibition than clopidogrel in patients after cardiac resuscitation with mild hypothermia [11,12,13]. In a meta-analysis of 1100 patients with CS or cardiac arrest, newer P2Y_12_ receptor inhibitors were associated with lower mortality rates without differences in bleeding risk [14].

Because of the aforementioned limitations of delayed onset of action and interindividual variability, clopidogrel has been largely replaced by the newer P2Y_12_ receptor inhibitors prasugrel and ticagrelor in the setting of ACS. Results from retrospective analyses and pharmacological data also support the use of the newer P2Y_12_ receptor inhibitors in the setting of CS. Thus, the use of clopidogrel in patients with CS, where an immediate onset of action is required, appears to be limited. However, clopidogrel has the lowest bleeding risk of all the P2Y_12_ receptor inhibitors and may therefore play an important role in the later phase of treatment in patients at high risk of bleeding or in combination with oral anticoagulants. All oral P2Y_12_ receptor inhibitors can be administered by nasogastric tube after crushing. Crushing may also be useful in patients without a nasogastric tube if a faster onset of action is desired, as shown in pharmacological studies [15,16]. A delayed onset of platelet inhibition was observed for all oral P2Y_12_ inhibitors in ACS/CS patients who were treated with opioids such as morphine and fentanyl, potentially increasing the risk of ischemic events [17,18,19]. These effects have been explained by impaired drug metabolism leading to reduced active drug concentrations due to reduced gastric emptying and delayed intestinal absorption rather than drug interactions on a pharmacological level [20,21,22]. Use of alternative analgesics (e.g., IV acetaminophen or peripheral opioid receptor antagonist) [23,24], coadministration of metoclopramide [25], crushed administration of oral P2Y_12_ receptor inhibitors, or use of intravenous platelet inhibitors might overcome attenuated platelet inhibition.

#### 3.2.4. Cangrelor

Cangrelor is a direct, reversible P2Y_12_ receptor inhibitor that blocks ADP-dependent platelet aggregation. It is administered intravenously as a bolus, followed by a continuous infusion. It inhibits platelets immediately after application. It is characterized by a very short plasmatic half-time of 3–6 min, the platelet function recovers completely within 30 min. The seamless transition from cangrelor to oral P2Y_12_ is of utmost importance to avoid gaps in platelet inhibition leading to thrombotic events/stent thrombosis. According to the drug label, a loading dose of clopidogrel, ticagrelor, or prasugrel should be administered immediately after cessation of the cangrelor infusion. Alternatively, a loading dose of ticagrelor or prasugrel, but not clopidogrel, can be administered 30 min before the end of the infusion [28]. The current ESC guidelines recommend that, in cases of concomitant ticagrelor treatment, ticagrelor (LD 180 mg) shouldbe administered at the time of PCI to minimize the potential gap in platelet inhibition during the transition phase [5].

Cangrelor was approved on the basis of large randomized trials comparing its effect with that of clopidogrel in patients with elective PCI and ACS [29,30,56,57]. Cangrelor was superior to clopidogrel for the combined endpoints of death, myocardial infarction, ischemia-driven revascularization, or stent thrombosis at 48 h after PCI. However, there are no large randomized trials comparing cangrelor with newer P2Y_12_ receptor inhibitors, and patients with CS were not included in the mentioned trials. Pharmacological studies show superior platelet inhibition with cangrelor even when newer oral P2Y_12_ receptor inhibitors are used in the setting of ACS [31,32,33]. In contrast to clinical trials, PCI in CS is one of the most common indications in real-world registries. In one retrospective analysis, cangrelor was associated with a better post-procedural TIMI flow grade; another retrospective analysis showed no difference in stent thrombosis in STEMI patients with cardiac arrest [34,35]. Data from a small RCT and a national registry suggest that cangrelor prevents ischemic complications in the early hours after PCI in CS patients and is more effective than oral P2Y_12_ inhibitors with an acceptable safety profile [36,37]. The limitation of cangrelor is a possible treatment gap during the transition to oral platelet inhibitors due to the very short half-time. However, pharmacologic studies showed constant platelet inhibition using early loading with both ticagrelor and prasugrel during the transition phase [39,40,41]. A larger randomized trial (DAPT-SHOCK, ClinicalTrials.gov Identifier: NCT03551964) is currently evaluating the effects of cangrelor versus crushed ticagrelor in CS patients undergoing PCI with the primary combined endpoint of death, myocardial infarction, and stroke.

Cangrelor was further investigated as bridging therapy (lower dose at 0.75 μg/kg per minute) in patients with prior ACS awaiting CABG Surgery (BRIDGE-trial) showing superior platelet inhibition with tolerable bleeding rates [38].

### 3.3. Glycoprotein IIb/IIIa Receptor Inhibitors (GPI)

GPIs inhibit platelets via the glycoprotein IIb/IIIa receptor, which mediates the binding of activated platelets to fibrinogen. There are two substances currently available: non-peptide antagonist tirofiban and the synthetic peptide eptifibatide. The monoclonal antibody abciximab has been withdrawn from the market. GPIs are administered as a bolus during coronary intervention, followed by a continuous infusion over 12–24 h. The dose is adjusted according to weight and renal function. They have an immediate onset of antiplatelet action and a plasmatic half-life of approximately 2–3 h. Stronger antiplatelet effects of tirofiban compared to chewed prasugrel and even cangrelor have been shown in the FABOLUS-FASTER trial, a pharmacodynamic study in STEMI patients [42].

According to current guidelines, GPIs are recommended as a bailout therapy during coronary intervention for ACS with thrombotic complications (e.g., high coronary thrombus burden, slow-flow/no-flow after stent implantation, stent thrombosis), as they are associated with increased bleeding events in combination with anticoagulants and oral antiplatelet therapy. In STEMI patients, routine use was associated with reduced mortality, but at the expense of bleeding [43]. Trial evidence supporting the use of GPIs in CS is still sparse. The PRAGUE-7 trial did not find a benefit of routine upstream use of abciximab compared to standard therapy; however, the small sample size limits definite conclusions. There are conflicting associations in registries, including CS patients treated with more potent oral antiplatelet compounds, reporting a mortality benefit under treatment with GPIs in some studies [44,45] and no benefit in others [46,47]. Potential drawbacks of GPIs are bleeding and the development of immune thrombocytopenia (ITP), which is a threat for long-term use in ICU patients, e.g., under ECMO. ITP with severe thrombocytopenia has been reported in clinical trials between 0.2–0.5% for tirofiban and eptifibatide [48,49,50].

### 3.4. Unfractionated Heparin (UFH)

UFH consists of a mixture of glycosaminoglycans of different lengths (15–150 hexose units). The anticoagulant effect is achieved by activating antithrombin III, which inhibits the coagulation factors Xa and thrombin. UFH is administered intravenously as a bolus or as a continuous infusion. It has a short half-life of about 1–2 h. Due to the inter-individual differences in metabolism and the narrow therapeutic window, anticoagulation is controlled by activated partial thromboplastin time (aPTT) or at higher doses by activated clotting time (ACT). In the event of bleeding, heparin can be antagonized with protamine. The occurrence of heparin-induced thrombocytopenia (HIT) should be mentioned as a possible side effect. UFH is administered as standard for anticoagulation during PCI (target ACT 250–350 s). It prevents the progression of an existing coronary thrombus and catheter-associated thromboembolic complications.

### 3.5. Low-Molecular-Weight Heparins (LMWHs) and Fondaparinux

LMWHs inhibit factor Xa in complex with antithrombin III and have a minimal direct effect on thrombin. They exhibit more predictable pharmacokinetics compared to unfractionated heparin (UFH) and are typically administered subcutaneously to patients requiring anticoagulation. Fondaparinux is a synthetic pentasaccharide that selectively inhibits Factor Xa. Like LMWH, it can be administered subcutaneously to patients with ACS. According to the current guidelines, it can be considered in patients with NSTEMI as a delayed invasive strategy, but is not recommended for STEMI patients [5,58]. Enoxaparin is the only LMWH approved for intravenous use during PCI in ACS patients. Due to the extended plasmatic half-lives of LMWH and fondaparinux, they are usually avoided in PCI for critically ill patients, and UFH is preferred.

There are no data from interventional trials comparing different anticoagulants and different dosing regimens guided by monitoring the anticoagulant effects of LMWH and UFH specifically in CS.

### 3.6. Bivalirudin

Bivalirudin is a synthetic hirudin derivative that inhibits thrombin selectively and independently of antithrombin III. It is administered periinterventionally and intravenously as a bolus and as a continuous infusion for up to 4 h in a weight-adapted manner in patients with ACS. Compared to heparin, bivalirudin is characterized by more predictable pharmacokinetics with less inter-individual variability. In randomized studies, patients taking bivalirudin showed a lower risk of bleeding than those taking heparin in combination with GPI. However, with selective use of GPI, bivalirudin showed no clear advantage and an increased risk of thrombotic complications (e.g., stent thrombosis) compared to heparin. Bivalirudin is therefore only offered as an alternative to heparin (e.g., in cases of heparin-induced thrombocytopenia or significant risk of bleeding) during percutaneous interventions. CS Patients requiring VA ECMO showed fewer thrombotic and bleeding events under bivalirudin compared to UFH treatment in one recent retrospective study [53].

### 3.7. Argatroban

Argatroban is a direct, reversible thrombin inhibitor. Similar to bivalirudin, it is administered intravenously as a bolus and continuous infusion. Its effect can be monitored by aPTT or ACT. Argatroban is a safe alternative in cases of heparin-induced thrombocytopenia (HIT) in patients requiring anticoagulation and during PCI. Some retrospective studies favor the use of argatroban, even in patients without HIT, during therapy with ECMO [54,55].

## 4. Antithrombotic Therapy in Special Situations (Figure 2)

### 4.1. Mechanical Circulatory Support (MCS)

#### 4.1.1. Extracorporeal Membrane Oxygenation (ECMO)

Patients on ECMO are jeopardized by thrombotic as well as bleeding complications that can occur simultaneously, making effective and safe antithrombotic therapy challenging. Blood is exposed to a large foreign surface area, including the membrane oxygenator, tubes, and pump. Adsorption of proteins involved in the coagulation cascade, exaggerated thrombin generation, and consumption of coagulation factors can foster procoagulatory and/or hyperfibrinolytic processes. UFH is most frequently used during ECMO to prevent clotting of the circuit. ACT of 180 to 200 s should be targeted. Of note, the ACT can be prolonged under hypothermia and be affected by coagulation factor levels. In addition, the correlation with other coagulation tests is inconsistent in a substantial number of cases [51]. Thus, for the management of anticoagulation, other laboratory parameters, including platelet count, hemoglobin, antithrombin levels, anti-Xa/heparin levels, and aPTT, should always be taken into consideration [52]. Viscoelastic assays (TEG-6S, ROTEM) using specific activators (e.g., tissue factor, ellagic acid, kaolin) provide complementary information to evaluate whole blood thrombogenicity and are often used for bleeding management. However, these assays have limitations in assessing the contribution of platelet reactivity in patients on dual antiplatelet therapy [59], and larger studies are warranted to establish trigger values for therapeutic decision making [60].

**Figure 2:**
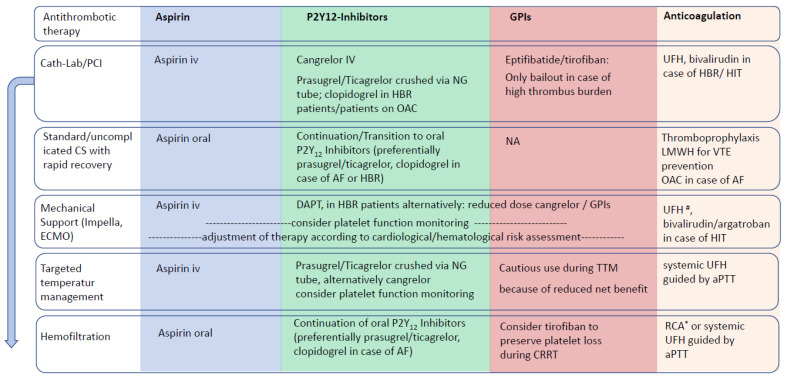
Cardiogenic shock—Aspects of Antithrombotic Therapy in Special Situations. # Calculate total UFH dose, including UFH purge concentration. * Contraindicated in liver failure and lactate acidosis. AF—atrial fibrillation; aPTT—activated partial thromboplastin time; CS—cardiogenic shock; DAPT—dual antiplatelet therapy; ECMO—extracorporeal membrane oxygenation; GPI—glycoprotein IIb/IIIa inhibitors; HBR—high bleeding risk; HIT—heparin-induced thrombocytopenia; MCS—mechanical circulatory support; NG—nasogastric tube; PCI—percutaneous coronary intervention; OAC—oral anticoagulation; RCA—regional citrate anticoagulation; TTM—targeted temperature management; UHF—unfractionated heparin.

Besides exaggerated anticoagulation, coagulation disorders can increase the bleeding risk under ECMO and should be carefully monitored. High shear stress and continuous flow of ECMO (as well as the Impella device) can induce proteolysis of von Willebrand factor, leading to acquired von Willebrand syndrome (avWS). Removal of the ECMO is currently the only solution for severe avWS, while preliminary data suggest the benefits of targeting ADAMTS13 [61]. Thrombocytopenia under ECMO is a relevant and challenging problem. Its causes and thus therapeutic considerations are manifold, including hemolysis, ineffective anticoagulation resulting in thrombosis in the vasculature and pump thrombosis, disseminated intravascular coagulation (DIC), HIT ideally detected by functional tests, sepsis, and/or bleeding. Factor XI inhibition targeting the contact activation pathway may be an attractive approach to reduce bleeding in ECMO patients in the future [62]. There is a lack of evidence on how to overcome thrombocytopenia. Treatable causes should be identified and addressed (e.g., switching from UFH to direct thrombin inhibitors such as argatroban or bivalirudin, anti-infectious therapy in the case of sepsis). Small studies suggest a benefit of cangrelor in cases of thrombocytopenia to counteract exaggerated immune complex-mediated platelet activation and further platelet consumption under ECMO [63,64]. Antiplatelet therapy in CS patients with previous PCI while on heparin anticoagulation represents a major challenge due to dynamic changes in the bleeding risk and potential vascular access site complications, making antiplatelet compounds with a long half-life undesirable. Nevertheless, in the experimental arm of the ECLS shock study, aspirin was given to 86.1%, clopidogrel to 27.2%, prasugrel to 49%, and ticagrelor to 22.3% of the patients. Moderate or severe bleeding occurred in 23.4% of ECLS patients [3]. There are only a few studies evaluating the use of short-acting IV antiplatelet drugs. Eptifibatide and cangrelor appear to prevent in-stent thrombosis but may increase bleeding in patients on ECMO in case reports [65,66]. Low-dose cangrelor (0.125 µg/kg/min starting dose continued for 5 days) combined with standard-intensity anticoagulation with bivalirudin was a feasible anti-thrombotic strategy in patients undergoing PCI during VA-ECMO support for ACS-related CS/CA. Still, major bleeding occurred in 21% of cases [67].

#### 4.1.2. Impella

Treatment with the microaxial flow pump (Impella, Abiomed, Danvers, MA, USA) requires anticoagulation via the purge solution containing up to 25,000 U/500 mL UFH and systemic anticoagulation with a starting dose of 11–12 U/kg bodyweight. The total UFH rate should not exceed 1800 U/h. As an example, a 95 kg patient with a purge rate of 10 mL/h and UFH purge concentration of 50 U/mL would require a total UFH infusion rate of 1140 U/h, resulting in a 500 U/h purge rate and the remaining 640 U/h by IV heparinization. UFH effects should be monitored with a target ACT of 160–180 s. Guiding according to factor Xa levels (window between 0.1 and 0.3 U/mL) has been associated with a low rate of thrombotic and bleeding complications [68]. In patients with HIT, alternative systemic anticoagulation with argatroban or bivalirudin and either an anticoagulant-free or alternative anticoagulant-containing purge solution is required. The dextrose concentration (either 5% or 20% dextrose) has an impact on the anticoagulatory effects of the purge solution. Thus, 5% dextrose runs through the Impella system more quickly, delivering more anticoagulant over time. This has to be taken into account for dosing of UFH and monitoring of anticoagulation effects [69].

### 4.2. Hemofiltration

Acute renal failure is common in CS and is associated with increased bleeding and thrombotic risk. Continuous venovenous hemofiltration (CVVH) with early initiation and longer duration was associated with improved outcomes in patients with CS and acute renal failure after cardiac surgery [70]. Regional citrate anticoagulation seems to be superior to UFH with regard to circuit life and bleeding reduction [71,72,73]. In a small randomized study, the addition of a small-molecule GPI tirofiban to UHF was studied in patients with CS requiring hemofitration. In comparison to anticoagulation with UHF alone, tirofiban significantly reduced platelet loss during continuous renal replacement therapy [74]. However, this small study was not powered for clinical endpoints such as mortality or bleeding.

### 4.3. Hypothermia/Targeted Temperature Management (TTM)

Hypothermia/targeted temperature management (32–34 °C) with or without VA-ECMO may attenuate the detrimental effects of ischemia reperfusion injury in cardiogenic shock, and may be associated with a mortality reduction and improved neurological outcome; however, the controversial results exist and the certainty of the evidence is relatively low [75,76,77,78,79]. Recent guidelines recommend continuous monitoring of core temperature and actively preventing fever for at least 72 h rather than enforcing hypothermia [78]. Hypothermia has a major impact on coagulation, fibrinolysis, platelet, and endothelial cell function [80,81]. Although it has a moderate effect on spontaneous platelet activity, it substantially influences agonist-induced platelet activation [82,83]. The higher risk for stent thrombosis reported in smaller studies [84,85] could not be confirmed in a large analysis containing 49,109 cardiac arrest patients, of whom 1155 underwent hypothermia [86]. The effects of P2Y_12_ inhibitors are attenuated under hypothermia [13], but prasugrel and ticagrelor still achieve sufficient platelet inhibition in contrast to clopidogrel [87,88]. Mild hypothermia (34 to 37 °C) augmented eptifibatide- and tirofiban-induced inhibition of platelet aggregation [89]. Cangrelor infusion prevented platelet activation and a subsequent increase in platelet count under hypothermia in an animal model as well as an in-vitro model of extracorporeal circulation (Chandler-loop) [60]. UFH dose adjustment is required in hypothermia with titration under tight control of aPTT and/or ACT.

## 5. Atrial Fibrillation

AF is more frequent in patients with ACS and CS (~20%) [90]. The presence of AF increases the risk of CS and contributes to the deterioration of hemodynamics. AF already present on admission was associated with increased mortality compared to new-onset AF, and the latter was not associated with increased mortality compared to non-AF CS patients [71,90]. Antithrombotic therapy for CS patients with concomitant AF is challenging. Patients with CS already on oral anticoagulation should receive an UFH bolus according to the ACS guidelines, i.e., if the patient is on a NOAC or if the INR is <2.5 in VKA-treated patients [5]. There is a lack of evidence regarding anticoagulation/antiplatelet combination therapy in CS patients with AF undergoing PCI. The risk for stent thrombosis is usually high, and there is consensus that effective antiplatelet therapy (i.e., DAPT) is essential during the first month [5,91,92,93]. However, there are situations of high bleeding risk, such as bleeding complications under ECMO, when individual treatment decisions are required. Thus, modifications of anticoagulant type and dose (dose reduction, switching from UFH to bivalirudin/argatroban) may be necessary in these patients.

## 6. Management of Antithrombotic Therapy in CS Patients with Bleeding

In cases of severe bleeding, heparin can be reversed by protamine sulfate. Management of bleeding should be tailored to every patient with the reversal of anticoagulation, transfusion (when the indication exists), and re-evaluation of the anticoagulation strategy. In patients undergoing MCS with bleeding complications, coagulation disorders (e.g., Factor II, V, VIII, X deficiency, HIT, DIC, hyperfibrinolysis, avWS) should be identified and treated accordingly under coagulation monitoring (e.g., using viscoelastic assays). The timing and resumption of antiplatelet therapy need to be judged on an individual, case-based, interdisciplinary decision [94].

## 7. Bridging to Destination Therapy/Surgery

There are few case series using short-half-life antiplatelet agents during bridging to surgery or ventricular assist device (VAD) in patients with ACS complicated by cardiogenic shock. In a series of patients under VA-ECMO treated with cangrelor (dose 0.75 µg/kg/min), hemorrhagic complications occurred frequently, with no recurrent coronary events observed [64]. Lowering the dose (i.e., 0.5 µg/kg/min) guided by platelet function monitoring was associated with lower bleeding rates without incidences of stent thrombosis in another case series of patients bridged to surgical procedures [95]. No specific guidelines exist regarding oral antiplatelet interruption/bridging protocol in CS patients undergoing coronary bypass surgery and/or LVAD therapy. Recommendations for preoperative antiplatelet management are to continue aspirin until the day of cardiac surgery and discontinue ticagrelor, clopidogrel, or prasugrel 3, 5, or 7 days before surgery, respectively, if the urgency of the indication allows [96].

## 8. Conclusions and Gaps in Evidence

Management of antithrombotic therapy remains a major challenge due to the dynamical change in thrombotic and bleeding risk and the altered pharmacodynamics of antithrombotic drugs in CS and organ failure. There is a lack of evidence from studies comparing different antithrombotic medication protocols in patients with CS, in particular those undergoing mechanical cardiac support. Also, larger randomized studies to evaluate the efficacy and safety of IV versus oral antiplatelet compounds in CS patients are warranted. An ongoing randomized trial is investigating the effects of cangrelor versus crushed ticagrelor in CS patients undergoing PCI (DAPT-SHOCK, ClinicalTrials.gov Identifier: NCT03551964). There is a lack of evidence regarding anticoagulation/antiplatelet combination therapy in CS patients with AF undergoing PCI. Systematic studies should further investigate anticoagulation protocols and give guidance on how to standardize hematological and hemostatic management in critically ill patients.

## Figures and Tables

**Table 1:** Pharmacological properties of different antithrombotic substances and special aspects in cardiogenic shock.

Drug	Route of Administration	Mode of Action	Plasma Half-life/Duration of Action since Last Dose	Dose	Pharmacokinetics/ Pharmacodynamics in CS	Particular Aspects in CS	References
Aspirin	Oral/IV	Irreversible COX-1 Inhibition	20 min/7–10 days	loading dose of 150–300 mg perorally or 75–250 intravenously; 75–100 mg daily maintenance dose	Platelet inhibition by oral aspirin may be reduced during TTM	IV preferred route	[5,6,7]
P2Y_12_ receptor inhibitors							
Clopidogrel	Oral/crushed	Irreversible P2Y_12_-Inhibition	30–60 min/3–10 days	600 mg loading dose; 75 mg daily maintenance dose	Delayed GI absorption Decreased hepatic metabolism	Due to two-step hepatic metabolism, conversion to active metabolite may be substantially impaired in CS patients with liver failure No sufficient antiplatelet effect in hypothermia Potential Interaction with CYP3A4, CYP3A5 or CYP2C19 inhibitors	[8,9,10,11,12,13,14,15,16,17,18,19,20,21,22,23,24,25,26,27]
Prasugrel	Oral/crushed	Irreversible P2Y_12_-Inhibition	30–60 min/7–10 days	60 mg loading dose; 10 mg daily maintenance dose (5 mg daily in patients >75 years of age)	Delayed GI absorption Decreased hepatic metabolism	Potential interactions with strong CYP3A4/A5 and CYP2B6 inhibitors Delayed onset with opioids Long offset of 7–10 days	
Ticagrelor	Oral/crushed	Reversible P2Y_12_-Inhibition	6–12 h/3–5 days	180 mg loading dose; 90 mg twice daily maintenance dose	Delayed GI absorption Decreased hepatic metabolism	Interactions with strong CYP3A4 inducers or inhibitors Delayed onset with opioids Contraindicated in liver failure	
Cangrelor	IV	Reversible P2Y_12_-Inhibition	3–6 min/1–2 h	Bolus of 30 µg/kg IV followed by 4 µg/kg/min infusion for at least 2 h or the duration of the procedure (whichever is longer) Bridge Dose: 0.75 µg/kg/min	Not dependent on hepatic/renal metabolism in CS	No drug interactions via CYP450 metabolism. No interaction with opiates	[28,29,30,31,32,33,34,35,36,37,38,39,40,41]
GP IIb/IIIa inhibitors							
Eptifibatide	IV	Blockade of the GP IIb/IIIa receptor	2–3 h/4 h	IV bolus of 180 µg/kg followed by a continuous infusion of 2 µg/kg/min 180 µg/kg by a continuous infusion dose of 1.0 µg/kg/min in patient with 30 ≤ CrCl < 50 mL/min)	Competitive inhibition of GP IIb/IIIa receptor Rapid recovery of platelet function	Cautious use during TTM because of reduced net benefit Clearance reduced in renal impairment. Contraindicated in thrombocytopenia (<100.000 cells/mm^3^), severe renal impairment (<30 mL/min)/renal dialysis and severe hepatic failure, patients with prior ICH, ischemic stroke within 30 days and prior fibrinolysis	[42,43,44,45,46,47,48,49,50]
Tirofiban	IV	Blockade of the GP Iib/IIIa receptor	1.5–2 h/4–8 h	Bolus of 25 µg/kg IV over 3 min, followed by an infusion of 0.15 µg/kg/min for up to 18 h. For CrCl ≤ 60 mL/min: LD, 25 µg/kg IV over 5 min followed by a maintenance infusion of 0.075 µg/kg/min continued for up to 18 h. Initial infusion rate of 0.4 μg/kg/min for 30 min followed by 0.1 µg/kg/min (CrCl < 30 mL/min use 0.05 µg/kg/min)		Cautious use during TTM because of reduced net benefit Contraindicated in thrombocytopenia (<100.000 cells/mm^3^), severe hepatic failure, patients with prior ICH, ischemic stroke within 30 days and prior fibrinolysis	
Anticoagulants							
UFH	IV	Thrombin inhibition and factor Xa inhibition by antithrombin complex formation/activation	1.5 h/2–6 h	IV bolus 70–100 U/kg during PCI. IV infusion titrated to achieve an aPTT of 60–80 s (or less) depending on further indications for anticoagulation (e.g., according to ECMO/Impella protocol)	Variable response in CS	In TTM, UFH dose adjustment/reduction required under frequent aPTT/ACT monitoring Can be antagonized by protamine in case of bleeding	[5,51,52]
LMWH (enoxaparin)	SC/IV	Factor Xa inhibition by antithrombin complex formation/activation, little effect on thrombin	4–8 h/12 h	Enoxaparin: During PCI: IV bolus of 0.3 mg/kg enoxaparin if the last s.c. administration was given > 8 h before balloon inflation	Less variability in drug response compared to UFH	Impaired subcutaneous absorption due to reduced tissue perfusion Monitoring of anti-Fxa activity may be necessary in critically ill patients/patients with acute renal failure	[5]
Direct intravenous thrombin inhibitors							
Bivalirudin	IV	Reversible direct thrombin inhibitor	25 min/1 h	0.75 mg/kg IV bolus, followed by 1.75 mg/kg/h IV infusion for duration of procedure, extended duration for up 4 h after STEMI Renal impairment: No reduction of bolus dose Reduction of IV infusion dose CrCl < 30 mL/min: 1 mg/kg/h Hemodialysis: 0.25 mg/kg/h HIT (off-label): 0.15–0.2 mg/kg/h IV; adjust to aPTT 1.5–2.5 times baseline value ECMO (off-label): Individual aPTT/ACT guided protocols (e.g., [53])	No need to titrate dose No need for routine ACT monitoring	Alternative anticoagulant in HIT No clinically relevant drug–drug interactions reported Safe and efficacious alternative to UFH in CS/ECMO	[53]
Argatroban	IV	Reversible direct thrombin inhibitor	40–50 min/2–4 h	Initial dose 2 µg/kg/minute, dose adjustments according to aPTT and ACT	Rapid onset of action, fast reversibility of its anticoagulant effect, inhibition of clot-bound thrombin Hepatically cleared	Alternative anticoagulant in HIT No dosage adjustment in renal-impairment Contraindicated in patients with severe liver dysfunction Alternative to UHF in ECMO	[54,55]

ACT—activated clotting time; aPTT—activated partial thromboplastin time; CrCl—creatinine clearance; CS—cardiogenic shock; COX-1—cyclooxygenase 1; ECMO—extracorporeal membrane oxygenation; GI—gastrointestinal; GP Iib/IIIa—glycoprotein Iib/IIIa; IV—intravenous; LMWH—low-molecular-weight heparins; PCI—percutaneous coronary intervention; SC—subcutaneous; TTM—target temperature management; UHF—unfractionated heparin.
